# Evaluation of meniscal injury on magnetic resonance imaging and knee arthroscopy in patient with anterior cruciate ligament tear

**DOI:** 10.1051/sicotj/2024051

**Published:** 2024-12-12

**Authors:** Toan Thanh Vo, Duc Thien Nguyen, Nguyen Anh Dinh Le, Ky Hien Van Nguyen, Hiep Khanh Vuu, Tu Anh Le, Nguyen Ba Le Phan

**Affiliations:** University of Health Sciences, Vietnam National University Ho Chi Minh City Vietnam

**Keywords:** Anterior cruciate ligament, Magnetic resonance imaging, Knee, Arthroscopy, Meniscus

## Abstract

*Introduction*: Meniscal injuries often occur in association with anterior cruciate ligament (ACL) injury. Failure to detect meniscal tears in patients with ACL injuries can lead to more complex tears and make them more difficult to repair. *Objective*: To determine the degree of correlation between magnetic resonance imaging (MRI) and knee arthroscopy in diagnosing meniscal injuries in patients with ACL tears. *Methods*: A prospective descriptive study was conducted on 185 patients diagnosed with ACL tears through knee arthroscopy at Thong Nhat Hospital from April 2023 to April 2024. *Results*: The accuracy of MRI and its correlation with arthroscopy in detecting meniscal injuries is 69.2%, indicating a low degree of agreement between MRI and arthroscopy results. Diagnosis of meniscal injury location has an accuracy of 57.1%, indicating a minimal to low degree of agreement between MRI and arthroscopy results. Diagnosis of the injury region: Accuracy over 85%, with Kappa coefficients ranging from 0.3 to 0.59, *p* < 0.001. Diagnosis of the morphology of meniscal injuries: Accuracy over 89%, with Kappa coefficients ranging from 0.26 to 0.66, *p* < 0.001. *Conclusion*: There is a minimal to moderate correlation between MRI and arthroscopy in detecting, and diagnosing the location, region, and morphology of meniscal injuries in patients with ACL tears. Therefore, caution is advised when diagnosing meniscal injuries based solely on MRI findings in patients with ACL tears.

## Introduction

Meniscal injuries often accompany ACL injuries, with a rate of 55–80%, particularly high in those with multiple ACL injuries [1, 2]. If meniscal injuries go undetected in ACL tear patients, they may worsen over time, especially in the lateral meniscus. The presence of meniscal injury with an ACL tear increases the risk of osteoarthritis to 60–90% within 10–15 years, leading to poor surgical outcomes and early knee osteoarthritis [3–6]. To assess the diagnostic value of MRI versus knee arthroscopy for meniscal injuries in ACL tear patients, we conducted the study “Evaluation of Meniscal Injury on Magnetic Resonance Imaging and Knee Arthroscopy in Patients with Anterior Cruciate Ligament Tear”.

## Materials and methods

### Study subjects

The study included 185 patients diagnosed with anterior cruciate ligament injury by knee arthroscopy and underwent knee MRI at the Department of Orthopedic Surgery, Thong Nhat Hospital from April 2023 to April 2024.

### Study design

Retrospective – descriptive case series.

### Inclusion criteria

All patients of all ages and genders diagnosed with ACL tears through knee arthroscopy who underwent preoperative knee MRI at the Department of Orthopedic and Trauma Surgery, Thong Nhat Hospital, from April 2023 to April 2024 and agreed to participate in the study were included in the analysis.

### Exclusion criteria

Patients with a history of ACL reconstruction or other knee joint surgeries; patients with injuries to more than two knee ligaments; and patients who did not participate fully throughout the follow-up process (from admission to arthroscopic surgery of the injured knee).

### Data collection methods

See Figure 1.

**Figure 1:**
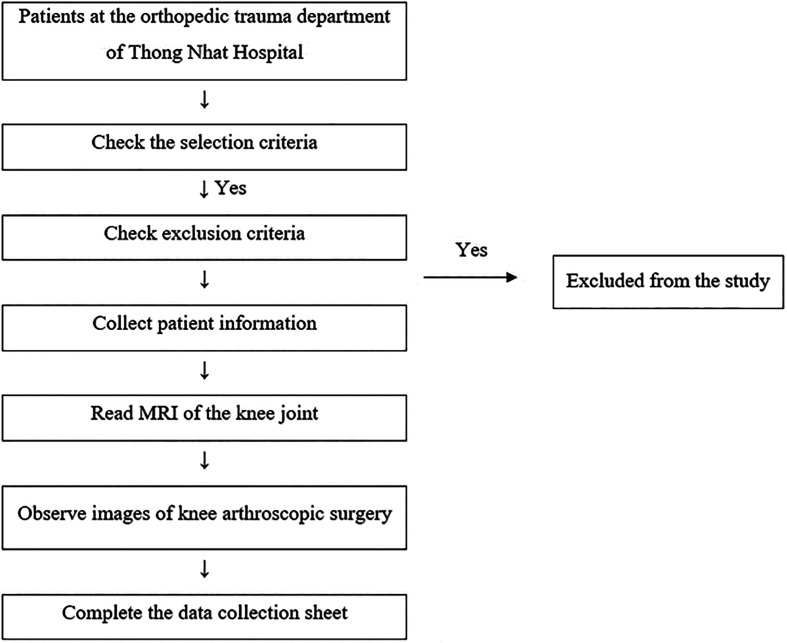
Sample selection process.

### Data processing methods

Quantitative variables following a normal distribution are expressed as mean and standard deviation; qualitative variables are presented as proportions. Categorical variables were tested using Chi-square and if the conditions were not met, Fisher’s exact test was used. Assessment of agreement between knee arthroscopy and MRI using Kappa test. Data were processed using SPSS software version 27.0.

## Results

### Study population

Out of the 185 patients included in this study, the majority were male (62.7%), with a higher occurrence of meniscal injuries in males (66.7%). Participants under 40 years old accounted for 58.9% of the study population, and 72.4% of the meniscal injuries occurred in this age group. The dominant leg was involved in 63.8% of cases, with 49.2% of meniscal injuries occurring in the dominant leg, compared to 43.3% in the non-dominant leg. Most injuries were acute (71.9%), but meniscal injuries were observed in 84.6% of patients with chronic injuries.

### Meniscal injury detection by MRI and arthroscopy

The overall accuracy of MRI for detecting meniscal injuries was 69.2%. However, a significant number of cases (approximately 25%) were false positives, demonstrating that MRI has limited diagnostic value for meniscal injuries in patients with ACL tears. The Kappa coefficient for agreement between MRI and arthroscopy was 0.4, indicating minimal agreement (*p* < 0.001) (Table 1).

**Table 1:** Value of MRI in diagnosing meniscal injuries (*n* = 185).

Meniscal injury on MRI	Meniscal injury on knee arthroscopy		SE	SPE	PPV	NPV	Kappa (*p*)
	*Yes*	*No*					
*Yes*	76	46	87.4%	53.1%	62.3%	82.5%	0.4
*No*	11	52					(<0.001)

NPV: Negative predictive values, PPV: Positive predictive values, SE: Sensitivity, SPE: Specificity.

### Severity of injury

When only grade 3 meniscus injuries were considered on MRI, the specificity increased to 80.5%, and the agreement between MRI and arthroscopy improved to a moderate level (Kappa = 0.44, *p* < 0.001). However, the sensitivity decreased to 72.2%.

### Meniscal injury location

In 104 cases (56.2%), MRI findings were completely consistent with arthroscopy, including 28 cases of medial meniscus injury, 14 cases of lateral meniscus injury, and 8 cases with injuries to both menisci. The remaining cases showed discrepancies between MRI and arthroscopy in terms of injury location, often involving the posterior horn of the medial meniscus. The Kappa coefficient for agreement in injury location was 0.34 for the medial meniscus and 0.5 for the lateral meniscus (*p* < 0.001) (Table 2).

**Table 2:** Correlation between location of meniscal injury on MRI and knee arthroscopy (*n* = 185).

Medial meniscus injury on MRI	Medial meniscus injury on arthroscopy		SE	SPE	PPV	NPV	Kappa
	*Yes*	*No*					
*Yes*	28	45	77.8%	69.8%	38.4%	92.9%	0.34
*No*	8	104					(<0.001)
Lateral meniscus Injury on MRI	Lateral meniscus injury on arthroscopy		SE	SPE	PPV	NPV	Kappa
	*Yes*	*No*					
*Yes*	15	8	46.9%	94.8%	65.2%	89.5%	0.5
*No*	17	145					(<0.001)

NPV: Negative predictive values, PPV: Positive predictive values, SE: Sensitivity, SPE: Specificity.

### Meniscal injury region

**Figure 2:**
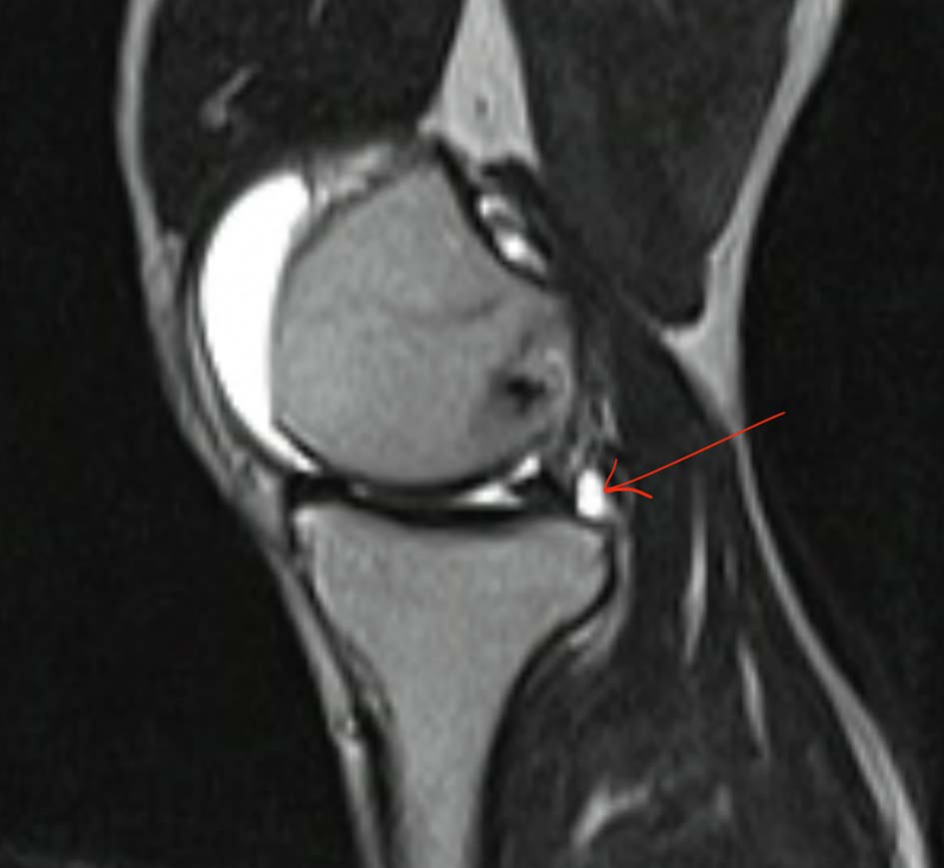
The MRI of the medial meniscus (the T2W MRI showed that the red arrow indicated a tear in the posterior horn of the medial meniscus).

**Figure 3:**
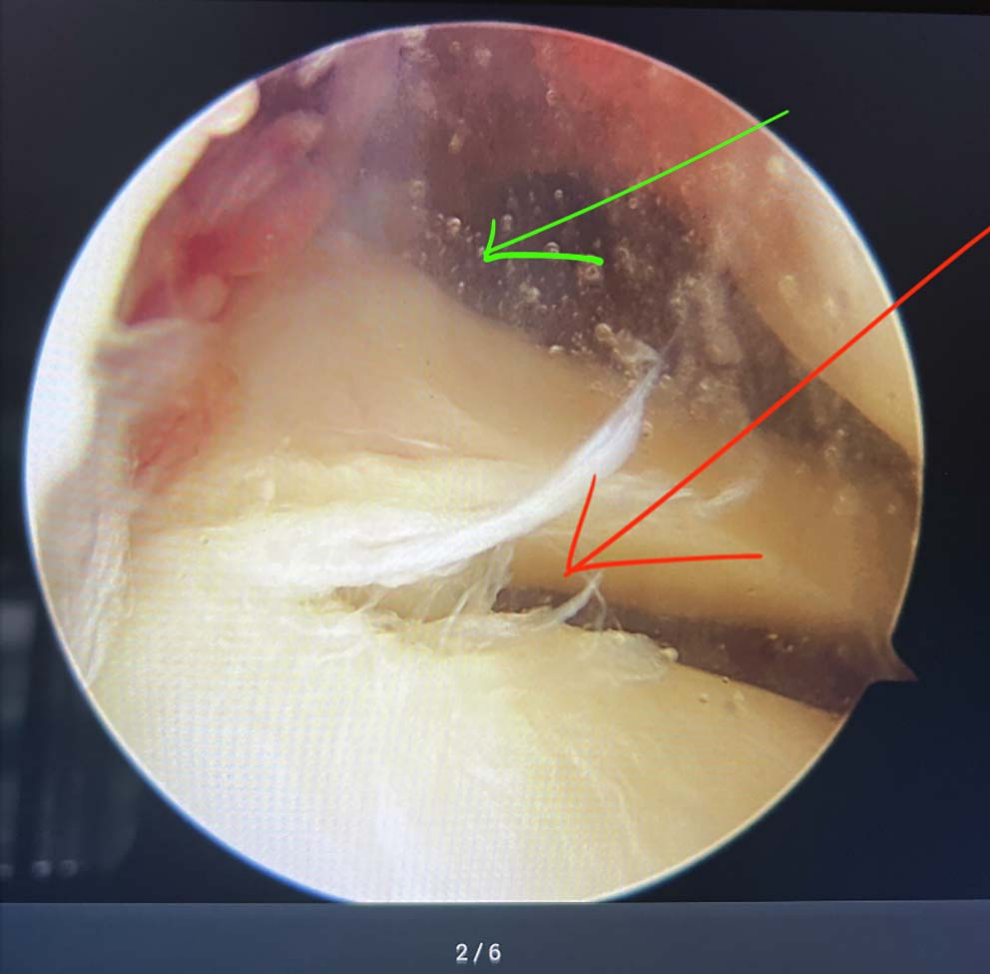
Arthroscopy of the medial meniscus (the red arrow indicated a degeneration in the body of the medial meniscus, the green arrow indicated a tear in the posterior horn of the medial meniscus).

MRI demonstrated varying sensitivities for detecting injuries in different regions of the meniscus. The sensitivity for detecting injuries in the anterior third and in more than two regions was 60%, while it was over 70% for the middle and posterior thirds. The Kappa coefficients ranged from 0.3 to 0.57, depending on the region (*p* < 0.001) (Table 3). For example: A 37-year-old male patient was diagnosed with a torn ACL in the left knee due to a sports injury. The patient underwent an MRI scan two weeks after the accident. After arranging his work, he was admitted to the hospital for arthroscopic ACL reconstruction surgery three months after the accident. There were similarities and dissimilarities between the MRI and an arthroscopic images regarding the area of the medial meniscus injury. The T2W MRI indicated a tear in the posterior horn of the medial meniscus (Figure 2) that resembles arthroscopy image, while arthroscopy revealed a degeneration in the body of the medial meniscus not shown in the MRI image (Figure 3).

**Table 3:** Diagnostic value of MRI for meniscus tear in ACL-injured knees (*n* = 185).

	SE	SPE	PPV	NPV	Kappa
**Morphology**					
Longitudinal	80%	88.4%	28.6%	98.7%	0.37
Horizontal	100%	91.9%	41.7%	100%	0.55
Radial	50%	97.9%	33.3%	98.9%	0.38
Vertical Flap	50%	97.7%	50%	97.7%	0.48
Horizontal flap	100%	97.8%	33.3%	100%	0.49
Complex	72.7%	93.8%	61.5%	96.2%	0.62
**Region**					
Anterior horn	60%	89.5%	25%	97.5%	0.30
Body	100%	92%	30%	100%	0.43
Posterior horn	71.4%	90.9%	58.8%	94.6%	0.57
More than two compartments	60%	91.9%	30%	97.5%	0.35

NPV: Negative predictive values, PPV: Positive predictive values, SE: Sensitivity, SPE: Specificity.

### Meniscal injury morphology

For medial meniscus injuries, MRI findings were consistent with arthroscopy in 61.6% of cases, resulting in a Kappa coefficient of 0.50 (*p* < 0.001). For lateral meniscus injuries, MRI results matched arthroscopy in 75.7% of cases, with a Kappa coefficient of 0.50 (*p* < 0.001). These findings suggest moderate agreement between MRI and arthroscopy in identifying the morphology of meniscal injuries (Table 3).

### Factors affecting the diagnostic results of meniscus injury on MRI

The diagnostic results of meniscus injury on MRI were influenced by several factors. Gender showed no significant correlation with meniscus injury, as indicated by a Chi-square test (*p* = 0.46). However, age was significantly correlated with meniscus injury (*p* = 0.02), as was the side of the injured leg, with a Kappa coefficient of 0.39 (*p* = 0.001). Additionally, the duration since the injury was strongly associated with a meniscus injury, as indicated by a Chi-square test (*p* < 0.001).

## Discussion

The results of this study indicate minimal to moderate agreement between MRI and knee arthroscopy in diagnosing meniscal injuries in patients with ACL tears. The overall accuracy of MRI was 69.2%, with a high false-positive rate, especially for low-grade meniscal injuries. These findings are consistent with previous studies, such as those by Gursoy et al. [7], which reported similar discrepancies between MRI and arthroscopy in ACL-injured patients. The table compared the sensitivity, specificity, and main findings of MRI in diagnosing meniscal injuries across different studies, providing a broader context for the study’s results (Table 4).

**Table 4:** Summary of the results of the most important published studies.

Author	Year	Number of patients	Sensitivity	Specificity	Main results
Gürsoy et al. [7]	2019	96	93.5% (MM),	88.8% (MM),	MRI accuracy is higher for medial meniscus than lateral meniscus and ACL injuries.
			64.8% (LM),	94.9% (LM),	
			55.5% (ACL)	81.6% (ACL)	
Nam et al. [8]	2014	50	88%	92%	MRI accuracy decreases in patients with ACL injuries, with lower sensitivity.
Phelan et al. [9]	2016	75	90%	85%	MRI is accurate for meniscal tears but less reliable for combined ACL injuries.
LaPrade et al. [10]	2015	100	95%	89%	High diagnostic value of MRI for meniscus root tears.
Van Dyck et al. [11]	2007	120	60%	80%	MRI shows low sensitivity for subtle meniscal injuries.
Kim et al. [12]	2021	60	67%	65%	MRI has limited diagnostic value for meniscus tear location.

One of the key challenges in using MRI to diagnose meniscal injuries in the presence of ACL tears is the interference caused by soft tissue swelling, effusion, and injury-related changes in the meniscal signal. This may explain the high rate of false positives observed in our study, particularly in the red-red zone of the meniscus, where injuries may have healed before arthroscopy but still appear on MRI. These findings align with the work of Van Dyck et al. [11], who reported that MRI sensitivity for subtle meniscal injuries was only 60%.

Additionally, the study demonstrated that MRI had limited accuracy in determining the location of meniscal injuries, especially in the posterior horn of the medial meniscus, where discrepancies between MRI and arthroscopy were frequent. This is consistent with the findings of Kim et al. [12], who also reported low sensitivity and specificity for detecting meniscal injury locations.

Interestingly, when only grade 3 meniscal injuries were considered, the diagnostic value of MRI improved, with specificity reaching 80.5%. This suggests that MRI may be more reliable for detecting more severe injuries, which are more likely to require surgical intervention. However, the lower sensitivity in detecting less severe injuries indicates that MRI should not be used as the sole diagnostic tool, especially in cases where the clinical suspicion of meniscal injury is high.

Furthermore, the study found that MRI had moderate agreement with arthroscopy in diagnosing the morphology of meniscal injuries, particularly for lateral meniscus injuries. These findings reflect previous studies, such as those by Dunn et al. [13], which also reported moderate interobserver agreement for MRI in classifying meniscal tear types.

In patients who did not undergo ACL reconstruction, the diagnostic accuracy of MRI for meniscal injuries may be even lower. Studies by Gursoy et al. (2019) [7] and Kim et al. (2021) [12] have shown that MRI is less reliable in diagnosing meniscal injuries when ACL injuries are not addressed, as the meniscal tears may progress over time. This progression can result in discrepancies between MRI findings and arthroscopic evaluations, especially in chronic cases where meniscal degeneration may occur between the time of the MRI and surgery.

The limitations of this study include its single-center design and the use of specific MRI equipment, which may have influenced the diagnostic accuracy. Additionally, the relatively small sample size and the exclusion of patients with isolated meniscal injuries may limit the generalizability of the findings. Future studies should aim to include a broader patient population and investigate the diagnostic value of MRI in patients without ACL tears.

### Study population

The study participants were predominantly male, likely due to their higher participation in high-contact sports, which increases their risk of injury compared to females, a finding consistent with other studies on knee injuries in Vietnam [14]. The age group under 40 had the highest participation rate (58.9%) and the highest rate of meniscal injuries (72.4%), as this group is more active in sports and labor, leading to a higher risk of knee injuries, including ACL and meniscus injuries, aligning with both domestic and international studies [15, 16]. The dominant leg was injured in 63.8% of cases, as its movements typically involve greater knee extension and hip flexion, making it more prone to sudden injuries [17]. Most participants (71.9%) presented with acute injuries, while the chronic injury group had a higher rate of meniscal injury (84.6%). These results are similar to those reported by Millett et al. (2002), who found that 36% of chronic ACL injury patients had meniscal injuries compared to 11% in the acute group [18].

### Meniscal injury detection by MRI and arthroscopy

The increase in false positives and decrease in specificity of MRI is due to the mechanism of injury involving the ACL. The impact force, whether valgus or varus, along with surrounding soft tissue effusion, changes the signal of the meniscus on MRI. Compared to the study by Gupta and colleagues (2023) [19], which also reported low specificity (<65%), this explains the false positive cases where meniscal injury locations on MRI often lie in the red-red zone of the meniscus, indicating a true injury that had fully recovered before surgery, leading to false positives on MRI.

### Severity of injury

Our study showed low positive predictive value for both medial and lateral meniscus injuries, differing from the study by Gursoy and colleagues (2019) [7]. The author also classified meniscal injuries on MRI into three grades, similar to our study, but considered grades 1 and 2 tears on MRI as normal meniscus, leading to high positive predictive value.

### Meniscal injury location

The high rate of false positives on MRI for location diagnosis is attributed to the anatomical features around the meniscus. The intermeniscal ligament pressing against the anterior horn of the lateral meniscus, the popliteal ligament passing through the posterolateral corner of the lateral meniscus, signal interference from part of the meniscus deflecting with the magnetic field (often in the posterior horn of the lateral meniscus), all contribute to false positives on MRI. The high rate of false negatives on MRI for lateral meniscus injury diagnosis is because patients with isolated ACL injuries may have subsequent meniscal injuries between the time of MRI and knee arthroscopy. Additionally, Van Dyck and colleagues (2007) [11] reported similar results with 60% sensitivity, suggesting that missed meniscal injuries on MRI are often related to subtle meniscal injury signs, reflecting the varying proficiency of MRI readers. Compared to the study by S. H. Kim (2021) [12], which also reported low sensitivity and specificity (67% and 65%, respectively) for diagnosing meniscal injury location, our study reflects similar findings for the posterior third of the lateral meniscus.

### Meniscal injury region

When compared to the study by S.H. Kim (2021) [12], our study showed a similar sensitivity of MRI in diagnosing the injury region of the medial meniscus (<70%). Especially for the anterior third of the meniscus, which is less frequently injured, explaining why this region is often missed [20]. However, our study differs from other studies where the Kappa coefficient for the anterior and middle thirds of the medial meniscus was minimal to low (0.3–0.43), while Chhabra (2019) [21] reported low to moderate (0.56–0.72). The Kappa coefficient was also similar to Chhabra’s study [8] for diagnosing the anterior and middle thirds of the lateral meniscus (0.58 and 0.43) with low agreement. However, our study differed for the posterior third of the lateral meniscus, showing low agreement. This discrepancy may be due to differences in equipment, MRI reading skills, and MRI acquisition techniques between the two studies [12, 21], as the criteria for classifying the anterior, middle, and posterior thirds are detailed and difficult to apply uniformly on MRI.

### Meniscal injury morphology

Regarding the morphology of meniscal injuries, 114 cases showed full agreement between MRI and arthroscopy results for medial meniscus injuries, accounting for 63.2%. The Kappa coefficient was 0.50 (*p*-value < 0.001). For lateral meniscus injuries, 140 cases showed full agreement, accounting for 76.92%, with a Kappa coefficient of 0.50 (*p*-value < 0.001). The overall diagnostic value of MRI for meniscal injury morphology was similar to previous studies, indicating its acceptance as a diagnostic tool. However, its diagnostic value for determining the specific type of meniscal injury was lower than the overall diagnostic value. Studies by Anderson et al. showed moderate to substantial interobserver agreement for MRI in identifying meniscal tear types, with Kappa values ranging from 0.46 to 0.72. Dunn et al. [13] also reported moderate to substantial agreement between MRI and surgical findings in classifying meniscal tear types and locations (Kappa 0.61–0.63). Prior studies evaluating the correlation between MRI findings and arthroscopy for meniscal tears using the ISAKOS classification indicated moderate agreement [22]. The results of this study align with previous studies regarding interobserver agreement and diagnostic value, including sensitivity, specificity, and accuracy. Therefore, MRI can be used as an acceptable diagnostic tool for detecting meniscal tears but may not be suitable for a detailed classification of meniscal tear types using the current ISAKOS standards [12].

### Factors affecting the diagnostic results of meniscus injury on MRI

Gender does not influence the rate of meniscal injuries, consistent with findings by Neil Ghodadra et al. (2012) [16]. The agreement between MRI and knee arthroscopy in detecting meniscal injuries varies by age, in line with studies by Tae-Seok Nam et al. (2014) and Seong Hwan Kim et al. (2021) [8, 12]. Injuries were more common in the dominant leg, which is more susceptible to both sudden and gradual injuries due to greater knee extension, hip flexion, and daily load-bearing. The time since injury significantly correlates with the rate of meniscal injuries (*p* < 0.001), with the higher agreement between MRI and knee arthroscopy in the chronic injury group (>8 weeks, Kappa = 0.50) compared to the acute group (≤8 weeks, Kappa = 0.28).

## Conclusion

There is a minimal to moderate correlation between MRI and knee arthroscopy in detecting, and diagnosing the location, region, and morphology of meniscal injuries in patients with ACL tears. Therefore, careful consideration is needed before concluding meniscal injuries based on MRI in patients with ACL tears.

## Data Availability

Corresponding authors take full responsibility for the data, analyses, and interpretation of the data, and for providing accurate data availability policies.

## References

[1] Kopf S, Beaufils P, Hirschmann MT, Rotigliano N, Ollivier M, Pereira H, Verdonk R, Darabos N, Ntagiopoulos P, Dejour D, Seil R, Becker R (2020) Management of traumatic meniscus tears: the 2019 ESSKA meniscus consensus. Knee Surg Sports Traumatol Arthrosc Off J ESSKA 28(4), 1177–1194. 10.1007/s00167-020-05847-3 PMC7148286 32052121

[2] Feucht MJ, Bigdon S, Bode G, Salzmann GM, Dovi-Akue D, Südkamp NP, Niemeyer P (2015) Associated tears of the lateral meniscus in anterior cruciate ligament injuries: risk factors for different tear patterns. J Orthop Surg Res 10, 34. 25889148 10.1186/s13018-015-0184-x PMC4389969

[3] Fok AW, Yau WP (2013) Delay in ACL reconstruction is associated with more severe and painful meniscal and chondral injuries. Knee Surg Sports Traumatol Arthrosc Off J ESSKA 21(4), 928–933. 10.1007/s00167-012-2027-1 22552616

[4] Kannus P, Järvinen M (1988) Knee ligament injuries in adolescents. Eight year follow-up of conservative management. J Bone Joint Surg Br Vol 70(5), 772–776. 10.1302/0301-620X.70B5.3192578 3192578

[5] Øiestad BE, Engebretsen L, Storheim K, Risberg MA (2009) Knee osteoarthritis after anterior cruciate ligament injury: a systematic review. Am J Sports Med 37(7), 1434–1443. 19567666 10.1177/0363546509338827

[6] Cohen M, Amaro JT, Ejnisman B, Carvalho RT, Nakano KK, Peccin MS, Teixeira R, Laurino CF, Abdalla RJ (2007) Anterior cruciate ligament reconstruction after 10 to 15 years: association between meniscectomy and osteoarthrosis. Arthrosc J Arthrosc Relat Surg Off Publ Arthrosc Assoc North Am Int Arthrosc Assoc 23(6), 629–634. 10.1016/j.arthro.2007.03.094 17560477

[7] Gürsoy M, Bulut T, Mete BD, Tosun Ö, Horoz EM, Gürsoy S (2019) The efficacy of magnetic resonance imaging in the determination of meniscus tears in patients with anterior cruciate ligament tears. Eastern J Med 24(2), 215–221.

[8] Nam TS, Kim MK, Ahn JH (2014) Efficacy of magnetic resonance imaging evaluation for meniscal tear in acute anterior cruciate ligament injuries. Arthrosc J Arthrosc Relat Surg Off Publ Arthrosc Assoc North Am Int Arthrosc Assoc 30(4), 475–482. 10.1016/j.arthro.2013.12.016 24680307

[9] Phelan N, Rowland P, Galvin R, O’Byrne JM (2016) A systematic review and meta-analysis of the diagnostic accuracy of MRI for suspected ACL and meniscal tears of the knee. Knee Surg Sports Traumatol Arthrosc 24(5), 1525–1539. 26614425 10.1007/s00167-015-3861-8

[10] LaPrade RF, Ho CP, James E, Crespo B, LaPrade CM, Matheny LM (2015) Diagnostic accuracy of 3.0 T magnetic resonance imaging for the detection of meniscus posterior root pathology. Knee Surg Sports Traumatol Arthrosc 23, 152–157. 25377189 10.1007/s00167-014-3395-5

[11] Van Dyck P, Gielen J, D’Anvers J, Vanhoenacker F, Dossche L, Van Gestel J, Parizel PM (2007) MR diagnosis of meniscal tears of the knee: analysis of error patterns. Arch Orthop Trauma Surg 127, 849–854. 17440743 10.1007/s00402-007-0318-7

[12] Kim SH, Lee HJ, Jang YH, Chun KJ, Park YB (2021) Diagnostic accuracy of magnetic resonance imaging in the detection of type and location of meniscus tears: comparison with arthroscopic findings. J Clin Med 10(4), 606. 33562787 10.3390/jcm10040606 PMC7914628

[13] Dunn WR, Wolf BR, Amendola A, Andrish JT, Kaeding C, Marx RG, Spindler KP (2004) Multirater agreement of arthroscopic meniscal lesions. Am J Sports Med 32(8), 1937–1940. 15572324 10.1177/0363546504264586

[14] Nguyen VN (2013) Comparing clinical diagnosis with magnetic resonance with endoscopy of damage to the meniscus and cruciate ligament of the knee joint. Master thesis, University of Medicine and Pharmacy at Ho Chi Minh City.

[15] Phung AT, Hoang TXM (2022) The value of MRI for determining meniscal tears in patients with injured knee. VMJ 521, 2.

[16] Ghodadra N, Mall NA, Karas V, Grumet RC, Kirk S, McNickle AG, Bach Jr BR (2013) Articular and meniscal pathology associated with primary anterior cruciate ligament reconstruction. J Knee Surg 26(3), 185–194. 23288741 10.1055/s-0032-1327450

[17] Caldwell Jr GL, Allen AA, Fu FH (1994) Functional anatomy and biomechanics of the meniscus. Oper Tech Sports Med 2(3), 152–163.

[18] Millett PJ, Willis AA, Warren RF (2002) Associated injuries in pediatric and adolescent anterior cruciate ligament tears: does a delay in treatment increase the risk of meniscal tear? Arthrosc J Arthrosc Relat Surg 18(9), 955–959. 10.1053/jars.2002.36114 12426537

[19] Gupta S, Ashish BC, Chavan SK, Gupta P (2024) Meniscus root tear: extended classification and arthroscopic repair techniques. Arthrosc Tech 13(1), 102807. 38312875 10.1016/j.eats.2023.08.012 PMC10837777

[20] Bullough P, Goodfellow J (1968) The significance of the fine structure of articular cartilage. J Bone Joint Surg Br Vol 50(4), 852–857. 5706888

[21] Chhabra A, Ashikyan O, Hlis R, Cai A, Planchard K, Xi Y, Shah J (2019) The International Society of Arthroscopy, Knee Surgery and Orthopaedic Sports Medicine classification of knee meniscus tears: three-dimensional MRI and arthroscopy correlation. Eur Radiol 29, 6372–6384. 31115621 10.1007/s00330-019-06220-w

[22] Denti M (2016) Synthesis. In: Hulet C, Pereira H, Peretti G, Denti M (eds) Surgery of the meniscus, Springer, Berlin, Heidelberg.

